# Modified Sijunzi Decoction Inhibits Epithelial-Mesenchymal Transition of Non-Small Cell Lung Cancer by Attenuating AKT/GSK3β Pathway *in vitro* and *in vivo*

**DOI:** 10.3389/fphar.2021.821567

**Published:** 2022-01-17

**Authors:** Niu Shao, Yao Xiao, Jiaxin Zhang, Yuying Zhu, Shenglong Wang, Suzhen Bao

**Affiliations:** ^1^ College of Basic Medical Science, Zhejiang Chinese Medical University, Hangzhou, China; ^2^ The First College of Clinical Medicine, Zhejiang Chinese Medical University, Hangzhou, China

**Keywords:** non-small cell lung cancer, modified sijunzi decoction, apoptosis, epithelial-mesenchymal transition, Akt/GSK3β pathway

## Abstract

Modified Sijunzi Decoction (MSJZD) is an empirical prescription of Traditional Chinese Medicine (TCM) and has been corroborated to be effective in multiple human diseases, but its role in non-small cell lung cancer (NSCLC) is enigmatic. Here we mainly analyze the function and mechanism of MSJZD in NSCLC. In this study, we used a method that coupled ultra-performance liquid chromatography to quadrupole time-of-flight mass spectrometry (UPLC-Q-TOF-MS) to investigate the major constituents in MSJZD with positive and negative ion modes. Additionally, in *in vitro* experiments, the effects of serum-containing MSJZD on the biological behavior of NSCLC cells induced by TGF-β1 were assessed by cell function experiments. Then, the influences of serum-containing MSJZD on epithelial-mesenchymal transition (EMT)-related markers were examined by immunofluorescence and western blot assays. Also, the AKT/GSK3β pathway and apoptosis-related markers were estimated by western blotting. Tumor xenografts were generated by subcutaneously injecting A549 cells into BALB/c nude mice to determine the effects of MSJZD *in vivo*. We first analyzed the composition of MSJZD. In positive ion mode, 47 kinds of components were identified. In negative ion mode, 45 kinds of components were identified. We also found that TGF-β1 contributed to inducing cell morphological changes and EMT progression. *In vitro*, surprisingly, cell proliferation, migration as well as invasion in NSCLC cells induced by TGF-β1, could be weakened by serum-containing MSJZD, and apoptosis was intensified. Moreover, serum-containing MSJZD weakened EMT passage and AKT/GSK3β pathway activation and induced apoptosis-related markers in NSCLC cells triggered by TGF-β1. *In vivo*, we discovered that MSJZD attenuated the tumor growth, promoted histopathological damage, and induced apoptosis in A549 tumor-bearing mice. Importantly, MSJZD has also restrained the development of EMT, AKT/GSK3β pathway, and TGF-β1 expression levels in nude mice. These findings demonstrated that MSJZD significantly weakened NSCLC progression by modulating EMT and AKT/GSK3β pathway.

## Introduction

Lung cancer is the most common malignant tumor in the world and its morbidity and mortality rate remain high (Adjei, 2019; Sharma et al., 2019). According to the 2020 Global Cancer Statistics report released by the International Agency for Research on Cancer (IARC), there were about 1.8 million deaths and 2.2 million new cases of lung cancer worldwide in 2020, accounting for 18% of the deaths and 11.4% of the new cases of malignant tumors, ranking first and second among all malignant tumors (Sung et al., 2021). Non-small cell lung cancer (NSCLC) accounts for about 80% of all lung cancer cases, about 75% of patients are already in the advanced stage when discovered, and the 5-years survival rate is only 15% (Wakelee et al., 2014). Currently, the treatment of NSCLC includes surgery, radiotherapy, chemotherapy, molecular targeted therapy, and immunotherapy, etc (Ruiz et al., 2014; Hirsch et al., 2016; Gadgeel, 2017; Allen et al., 2018; Yang and Mu, 2018). Although the above treatments have certain effects, distant metastasis in most patients is still the cause of the high mortality rate of NSCLC (Du et al., 2016). Therefore, it is of great significance to research the invasion and metastasis of tumors for the treatment of NSCLC.

Epithelial-mesenchymal transition (EMT) is a key event in the process of early tumor metastasis and progression. A variety of tumor cells can lose the epithelial cell-like phenotypic characteristics such as cell-to-cell contact and cell polarity through EMT, thereby gaining invasion and migration capabilities (Aiello et al., 2017; Ye et al., 2017). Transforming growth factor-β1 (TGF-β1) is a multifunctional cell regulator, which plays a bidirectional role in the development of cancer. TGF-β1 was overexpressed in NSCLC tissues, the prognosis of patients with overexpression of TGF-β1 was poor, and its high expression may indicate the progression or metastasis of NSCLC (Huang et al., 2014). Meanwhile, as one of the earliest multifunctional cytokines that can induce EMT, TGF-β1 can participate in a variety of biological processes by inducing EMT, such as fibrotic diseases, embryonic development, and cancer (Yang and Weinberg, 2008). Consequently, it is of major significance to study the mechanism of NSCLC metastasis and investigate effective drugs against NSCLC metastasis.

Traditional Chinese medicine (TCM) is rich in bioactive components, which play a role by targeting multiple molecular networks associated with the disease. Whereas, TCM is a potential candidate drug that can be developed for the prevention and treatment of cancer. In recent years, TCM has shown unique advantages and curative effects in many aspects of lung cancer treatment, such as reducing the toxic and side effects of radiotherapy and chemotherapy, adjusting the immune function of the body, stabilizing tumor foci, etc. (Jiang et al., 2019; Xu et al., 2019). Scientific research proved that single TCM extracts, such as *Astragali Radix*, and *Poria*, have certain effects on inducing lung cancer cell apoptosis, inhibiting cell invasion, metastasis, and angiogenesis (Cheng et al., 2014; Lee et al., 2018; Xu et al., 2018; Lin et al., 2020). Modified Sijunzi Decoction (MSJZD) was composed of *Astragali Radix*, *Poria*, *Codonopsis Radix*, *Rhizoma Atractylodis Macrocephalae*, *Pinelliae Rhizoma*, *Fritillariae Thunbergii Bulbus*, *Herba Hedyotis*, and *Glycyrrhizae Praeparata cum Melle Radix et Rhizoma*. The most basic theory of TCM in the treatment of NSCLC is regulating the deficiency of body energy to inhibit tumor cell growth, proliferation, invasion, and migration (Li et al., 2021). MSJZD is a TCM prescription as adjuvant therapy in the standard treatment of NSCLC, which is commonly used clinically for patients with lung- and spleen-Qi deficiency. Additionally, modern studies have found that deficiency of spleen-Qi is also closely associated with the body’s anti-cancer immunity (Li, 2007; Li et al., 2021). Accumulated studies have demonstrated the nourish spleen-Qi effect of SJZD in chemotherapy-induced immunotoxicity and on immune function in post-operative patients (Guan et al., 2018; Chen et al., 2019). According to TCM’s theory, the spleen contributes to the production of sputum, as well as lung stores the sputum; Thus, phlegmatic hygrosis also affects the metastasis of lung cancer in patients with lung- and spleen-Qi deficiency. Because of this, the MSJZD used mentioned above used Sijunzi decoction as a major formula and combined it with the *Pinelliae rhizoma*, *Fritillariae thunbergii* Bulbus, *Herba hedyotis*, and *Astragali Radix*, that possess synergistic or additive activity to promote the invigorating spleen and replenishing qi of the Sijunzi decoction. Besides, Many components of MSJZD have good clinical effects on mitigating cancer-related symptoms. For example, Quercetin, Liquiritigenin, Peimine, and Liquiritin all manifested good anti-tumor proliferation and metastasis effects (Wei et al., 2017; Meng and Lin, 2019; Reyes-Farias and Carrasco-Pozo, 2019; Tan et al., 2020). Although the clinical efficacy of MSJZD in the treatment of lung cancer metastasis has been confirmed, however, its therapeutic mechanism is still ambiguous.

In this study, we start with MSJZD as a whole and further evaluate the anti-tumor effect of MSJZD on EMT in lung cancer by preparing serum-containing MSJZD and establishing an A549 cell tumor-bearing model in mice, to provide a scientific basis for guiding clinical treatment.

## Materials and Methods

### Ethics Statement

The animal programs complied with the guidelines of the China Animal Care and Use Committee. Approval was acquired from the Committee of Laboratory Animals of Zhejiang Chinese Medical University Laboratory Animal Research Center (License number: SYXK (Zhe) 2018-0012). All efforts were made to alleviate the suffering of animals.

### Preparation Aqueous Extract of MSJZD

*Codonopsis Radix* 15 g, *Astragali Radix* 30 g, *Rhizoma Atractylodis Macrocephalae* 20 g, *Poria* 15 g, *Pinelliae Rhizoma* 12 g, *Fritillariae Thunbergii Bulbus* 12 g, *Herba Hedyotis* 30 g, and *Glycyrrhizae Praeparata cum Melle Radix et Rhizoma* 10 g were actually weighed and soaked in 1500 ml of distilled water for 60 min (min), then boiled at 100°C for 45 min. After harvesting the decoction solution, another 1200 ml of distilled water was added for the second extraction at 100°C for 30 min. After combining the filtrates, the decoction solution was concentrated to 100 ml with a final concentration of 2.8 g/ ml. The 2.8 g/ ml MSJZD solution was diluted with ultrapure water to 0.288 g/ ml aqueous extract of MSJZD. Finally, 0.5 ml MSJZD solution (0.288 g/ ml) was diluted to 0.144 g/ ml test solution with 0.5 ml methanol for UPLC-Q-TOF-MS analysis.

### UPLC-Q-TOF-MS Analysis

The aqueous extract of the MSJZD sample was analyzed on a Waters ACQUITY UPLC I-Class PLUS system (Waters Corporation, Milford, MA, United States) equipped with a Waters UPLC BEH C18 column (100 mm × 2.1 mm, 1.7 µm particle size) at a column temperature of 40°C. The mobile phase consisted of acetonitrile (A) and water (B), each containing 0.1% formic acid. The elution procedure was as follows: 99–99% B at 0–1 min; 99%~50% B at 1–15 min; 50%~40% B at 15–17 min; 40%~1% B at 17–18 min; 1% B at 18–21 min. The flow rate was 0.3 ml/ min, and the injection volume was 2 μL.

The mass spectrometric data were collected using a time-of-flight analyzer with TurboIonSpray ion source in both positive and negative ion modes. The specific conditions were as follows: nebulizing gas (N2): 55 psi; drying gas (N2): 45 psi; curtain gas (CUR): 35 psi; source temperature: 600°C; ions apart voltage floating (ISVF): 5500 V/-4500 V; TOF MS scan m/z range: 50–1500 Da; TOF-MS/MS scan m/z range: 25–1000 Da; TOF MS scan accumulation time: 0.25 s/spectra; product ion scan accumulation time: 0.035 s/spectra. Secondary mass spectrometry was obtained by information Dependent Acquisition (IDA) and high sensitivity mode. Declustering potential (DP) was ±60 V (two modes of positive and negative ions); collision energy was 35 ± 15 eV; IDA setup was as follows: Exclude isotopes within 4 Da; candidate ions to monitor per cycle was 12. The data were processed using SCIEX OS software with multiple confidence criteria, including quality accuracy, retention time, isotopes, and matching use of compound libraries. In the current study, the TCM MS/MS Library in the SCIEX OS software was employed to identify the major constituents in MSJZD according to the first-order accurate mass number, isotope distribution ratio, and MS/MS of the constituents.

### Preparation of Serum-Containing MSJZD

A total of 40 male Sprague-Dawley (SD) rats (SPF grade, weighting 200 ± 10 g) were purchased from Shanghai SLAC Laboratory Animal Co., Ltd [(SCXK (Hu) 2017-0005, Shanghai, China)]. All rats were raised in the Animal Center of Zhejiang Chinese Medical University with a light- and temperature-controlled room (room temperature, 20–25°C; relative humidity, 40–60%, 12/12 h light/dark cycle), and received ad libitum access to food and tap water. Before the experiment, the rats were adapted to the laboratory housing conditions for 1 week.

All animals were randomly divided into four groups with 10 rats in each group, namely the control group, MSJZD low-dose group, MSJZD medium-dose group, and MSJZD high-dose group. The rats in the MSJZD administration group were gavaged MSJZD at the dose of 7.5 g/ kg (0.5 times clinical equivalent dose), 15 g/ kg (clinical equivalent dose), and 30 g/kg (2 times clinical equivalent dose), respectively for 5 days. The control group was fed with normal saline at 1 ml per 100 g body weight for five consecutive days. The drug clinical equivalent dose conversion formula was as follows: (human dose of crude herbs in clinic /60 kg)×6.3. They were administered MSJZD twice a day for 5 days with an interval of 10 h. After the first MSJZD intragastric administration of rats on the 5^th^ day for 1 h, we utilized pentobarbital sodium (40 mg/ kg intraperitoneally, bm-007, Merck, Germany) to anesthetize rats (Mao et al., 2015). Blood samples were harvested through the abdominal aorta and left at room temperature for 0.5 h and then centrifuged at 3500 r/min for 20 min. The upper serum was the serum-containing MSJZD which was then inactivated at a 56°C water bath for 30 min. Then, filter the serum using a syringe filter with a 0.22-µm pore size hydrophilic polyethersulfone membrane. After filtration, the filtrate was harvested and stored at -20°C for subsequent cell experiments.

### Cell Lines and Cell Culture

Human NSCLC cell lines, A549, and H1299 were obtained from the American type culture collection (United States), and cultured in DMEM medium (SH30243.01, Hyclone, United States) with 10% fetal bovine serum (FBS) (11011-8615, Tianhang, China) and 1% penicillin-streptomycin (HyClone, Logan, Utah, United States). Also, the A549 and H1299 cells were grown as a monolayer in a cell incubator (BB150, ThermoFisher, United States) with standard culture conditions (5% CO_2_, 37°C, 95% air atmosphere).

### Experimental Design

We first utilized 5 ng/ ml TGF-β1 (RP00161, ABclonal, United States) to treat cells for 0, 24, 48, and 72 h and observed the alterations of cellular morphology and EMT-related markers at 72 h. Then, to check the roles of serum-containing MSJZD on cells, the cells were assigned to the blank group (cells were exposed to 10% control rat serum), TGF-β1 group (cells were subjected to 5 ng/ ml TGF-β1 and 10% control serum), low-dose group (cells were subjected to 5 ng/ ml TGF-β1 and low-dose serum-containing 10% MSJZD), medium-dose group (cells were stimulated with 5 ng/ ml TGF-β1 and medium-dose serum-containing 10% MSJZD), and high-dose group (cells were exposed to 5 ng/ ml TGF-β1 and high-dose serum-containing 10% MSJZD).

### Cell Viability

Cells (2×10^4^/well) were grown as a monolayer on 96-well plates for 24 h. Then, cells were processed according to the mentioned experimental design. After that, cells were exposed to 10 μL CCK-8 solution (HY-K0301, MCE, United States) at 24, 48, and 72 h for another 1 h. In the end, the optical density of each well was measured by a spectrophotometer at a wavelength of 450 nm (EPOCH2, Biotek, United States).

### Apoptosis Assay

For the evaluation of apoptosis, the Annexin V-FITC kit (556547, BD Pharmingen, United States) was used in this study. After serum-containing MSJZD treatment, the centrifuged A549 and H1299 cells (1.5 × 10^6^/well) were re-suspended in 1 × binding buffer until the cell concentration was 1.2 × 10^6^/well. After centrifugation, the precipitated cells were incubated with 5 μL Annexin V-FITC at 37°C away from light for 10 min followed by reaction with 5 μL PI at 37°C away from light for 5 min. After adding 1 × binding buffer, we utilized a flow cytometer (CytoFLEX, Beckman Coulter, United States) to determine the apoptosis rate of A549 and H1299 cells.

### Transwell Assay

Cell migration was measured using inserts (3422, Corning, United States) without matrigel matrix, while cell invasion was carried out using inserts with matrigel matrix (356234, BD Biosciences, United States). The treated cells (1 × 10^4^/well) in 100 μL non-FBS medium were plated into the inserts and the lower compartment was filled with medium augmented with 10% FBS. After reacting for 24 h, the cells on top of the chambers were cleaned with Q-tips and cells on the lower compartment were fixed in 4% paraformaldehyde (DF0135, Leagene, China) for 15 min before staining with 0.1% crystal violet (548-62-9, Qiangshun, China) for 0.5 h. At last, the images were gained of the bottom side of the membrane by an optical microscope (AE 2000, Motic, Germany). The number of migrated and invaded cells were quantified by ImageJ 1.52a software.

### Wound Healing Analysis

In brief, cells at a concentration of 5 × 10^5^ per well were plated into the 6-well plates overnight at 37°C with 5% CO_2_. Subsequently, we utilized a 0.2 ml yellow sterile pipet tip to draw a gap. After reacting for 48 h at 37°C with 5% CO_2_, the results were observed and pictures were gained in the optical microscope at two-time points (0 and 48 h).

### Immunofluorescence

Cells at a concentration of 1 × 10^5^ per well were injected into the 6-well plates containing cover slips. After being subjected to the indicated treatments, each well was fixed with methanol followed by permeabilization with 0.5% Triton X-100. After sealing with 1% BSA for 30 min, each well was subjected to anti-E-cadherin antibody (1:500, ab40772, Abcam, United Kingdom), anti-Vimentin antibody (1:1000, ab92547, Abcam, United Kingdom), and anti-Fibronectin antibody (1:50, ab268020, Abcam, United Kingdom) at 4°C overnight. Following reaction with anti-rabbit secondary antibody for 1 h at 37°C, the nuclei were stained with DAPI, which was then captured by fluorescence microscopy (Ts2-FC, Nikon, Japan).

### Establishment of A549 Cell Tumor-Bearing Model in Mice

Thirty male BALB/c nude mice (SPF grade, weighing 16 ± 2 g, and 3–5 weeks) were obtained from Shanghai SLAC Laboratory Animal Co., Ltd (Shanghai, China), and raised in the Animal Center of Zhejiang Chinese Medical University in a specific pathogen-free (SPF) facility. These mice have free access to diet and water for 7 days to acclimate to the environment. A549 cell suspension (5 × 10^6^ in 100 μL sterilized PBS) was injected subcutaneously into the left armpit of the nude mice. When the tumor size reached nearly 50 mm^3^, they were randomly assigned to five groups, six mice in each group, namely model group, 11 g/ kg MSJZD group (0.5 times clinical equivalent dose), 22 g/ kg MSJZD group (clinical equivalent dose), 44 g/ kg MSJZD group (2 times clinical equivalent dose), and 3 mg/ kg Cisplatin group. The drug clinical equivalent dose conversion formula was as follows: (human dose of crude herbs in clinic /60 kg) × 9.1. The nude mice in the MSJZD group were gavaged with 11 g/kg, 22 g/kg, or 44 g/ kg MSJZD, respectively, every day until the 28^th^ day. The nude mice in the cisplatin group were injected intraperitoneally with 3 mg/ kg cisplatin once every 3 days. The nude mice in the model group were given an equal volume of normal saline once a day for 28 days.

### Detection of the Tumor Volume and Weight

The tumor volume of mice from all groups was measured and calculated every 7 days until the 28^th^ day, and the body weights of the mice were measured every 3 days during the experiment. The tumor volume (V) was calculated using the following formula: V = 0.5 × W^2^ × L (W: the width of the tumor; L: the length of the tumor). After the 28^th^ day, they were sacrificed using 35 mg/kg pentobarbital sodium, and simultaneously, the tumor tissues were aseptically stripped, weighed, and taken pictures. A portion of the tissues was fixed in 4% paraformaldehyde for H&E staining, TUNEL, and immunohistochemistry (IHC) assay. Other tissues were utilized for qRT-PCR and western blot assays.

### H&E Staining

The fixed tissues were dehydrated, which were then embedded in paraffin. After cutting into 5 μm sections, the sections were dewaxed and hydrated followed by coloration with hematoxylin staining solution (G1004, Servicebio, China). After the sections were differentiated and washed, they were placed in 1% eosin solution (C0109, Beyotime, China) for 5 min followed by dehydration and transparency. In the end, images were observed and taken using the optical microscope after sealing the sections.

### TUNEL Staining

TUNEL Apoptosis Assay Kit (C1086, provided by Beyotime, China) was utilized in this research. The paraffin sections were subjected to treatment with dewaxing and rehydration followed by a reaction with DNase-free proteinase K (ST532, Beyotime, China) at 37°C for 20 min. After washing thoroughly, the sections were exposed to the prepared TUNEL detection solution at 37°C away from light for 1 h. After washing, the sections were blocked with Antifade Mounting Medium with DAPI (P0131, Beyotime, China) and then observed in the fluorescence microscopy with its excitation and emission wavelength at 500 and 565 nm, respectively.

### IHC Assay

The paraffin sections were dewaxed, rehydrated, and then rinsed with PBS. After antigen repair, the sections were subjected to 3% hydrogen peroxide solution in the dark at 37°C for 25 min. After blocking, they were subjected to anti-E-cadherin antibody (1:500), anti-Vimentin antibody (1:500), anti-TGF-β1 antibody (1:500, ab215715, Abcam, United Kingdom), and anti-Ki67 antibody (1:200, ab16667, Abcam, United Kingdom) at 37°C overnight. The next day, the sections were exposed to a secondary antibody at 37°C for 1 h, which were then developed using DAB (abs9210, absin, China). After dehydration, we utilized a mounting medium to seal the sections. Lastly, images were observed and obtained under the optical microscope.

### Quantitative Real-Time PCR

RNA from tissues was got using Trizol reagent (abs60031, absin, China). Afterward, the qRT-PCR reaction was conducted by TaqMan One-Step RT-qPCR Kit (T2210, Solarbio, China) in an EDC-810 PCR system (Eastwin Life Sciences, Inc.) under the manufacturer’s instructions. β-actin was taken as the normalization control. The 2^−ΔΔCT^ was taken as count the relative expressions of the gene (Sun et al., 2020). The primers were as follows: E-cadherin forward: 5′-AAA​GGC​CCA​TTT​CCT​AAA​AAC​CT-3′, E-cadherin reverse: 5′-TGC​GTT​CTC​TAT​CCA​GAG​GCT-3’; Vimentin forward: 5′-TGC​CGT​TGA​AGC​TGC​TAA​CTA-3′, Vimentin reverse: 5′-CCA​GAG​GGA​GTG​AAT​CCA​GAT​TA -3’; Snail forward: 5′-ACT​GCA​ACA​AGG​AAT​ACC​TCA​G-3′, Snail reverse: 5′-GCA​CTG​GTA​CTT​CTT​GAC​ATC​TG-3’; TGF-β1 forward: 5′-CTA​ATG​GTG​GAA​ACC​CAC​AAC​G-3′, TGF-β1 reverse: 5′-TAT​CGC​CAG​GAA​TTG​TTG​CTG-3’; AKT forward: 5′-AGC​GAC​GTG​GCT​ATT​GTG​AAG-3′, AKT reverse: 5′-GCC​ATC​ATT​CTT​GAG​GAG​GAA​GT-3’; GSK3β forward: 5′-GAC​TAA​GGT​CTT​CCG​ACC​CC--3′, GSK3β reverse: 5′-TTA​GCA​TCT​GAG​CTC​TGC​TGT-3’; β-actin forward: 5′-GGA​GCG​AGA​TCC​CTC​CAA​AAT-3′, β-actin reverse: 5′-GGC​TGT​TGT​CAT​ACT​TCT​CAT​GG-3’.

### Western Blot

After treatment, cells and tissues were treated with RIPA buffer (P0013D, Beyotime, China) containing PMSF (ST506, Beyotime, China) to acquire total proteins followed by quantification with BCA kit (pc0020, Solarbio, China). After denaturation and electrophoresis, the protein was transferred to a nitrocellulose membrane. After blocking, the nitrocellulose membranes were exposed to specific primary antibodies overnight at 4°C. The next day, the bound antibodies were subjected to an anti-rabbit secondary antibody for 90 min at 37°C. In the end, signals were examined by an ECL reagent (abs920, absin, China) with ChemiScope 3300 mini equipment (Clinx, China). The primary antibodies of anti-E-cadherin antibody (1:40000), anti-Vimentin antibody (1:5000), anti-Fibronectin antibody (1:1000), anti-Snail antibody (1:1000, ab216347), anti-AKT antibody (1:500, ab8805), anti-p-AKT antibody (1:1000, ab38449), anti-GSK3β antibody (1:8000, ab32391), anti-p-GSK3β antibody (1:10000, ab75814), anti-Bax antibody (1:2000, ab182733), anti-Bcl-2 antibody (1:1000, ab32124), anti-β-actin antibody (1:200, ab115777) were obtained from Abcam (United Kingdom).

### Statistical Analysis

Each experiment was repeated independently at least three times. Data were analyzed using Graphpad Prism 8.0 (GraphPad Software Inc., United States). All data were revealed as mean ± standard deviation. One-way ANOVA followed by SNK’s multiple comparison test was utilized when data followed a normal distribution. Kruskal-Wallis H test was utilized when data did not follow a normal distribution. A statistically significant difference was exhibited as *p < 0.05*.

## Results

### Total Ion Chromatogram of Modified Sijunzi Decoction (MSJZD) Obtained by UPLC/Q-TOF MS Analysis

**FIGURE 1:**
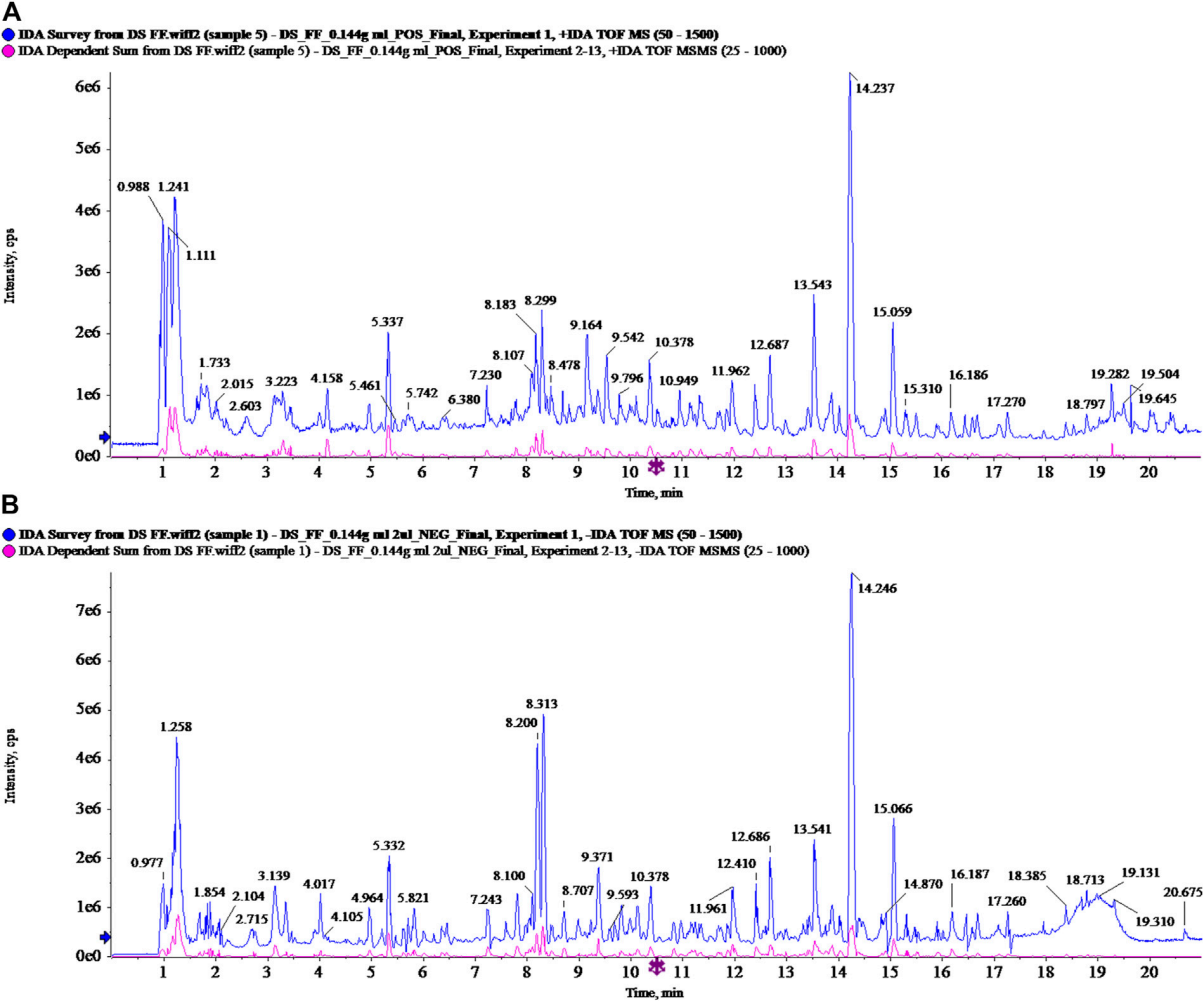
Total ion chromatogram of Modified Sijunzi Decoction (MSJZD) obtained by UPLC/Q-TOF MS analysis in **(A)** positive ion mode and **(B)** negative ion mode.

The MSJZD test solution was firstly analyzed by the UPLC-Q/TOF-MS system. Figure 1 manifested the total ion chromatogram of MSJZD in (A) positive ion mode and (B) negative ion mode. In positive ion mode, 47 kinds of components were identified (Table 1). In negative ion mode, 45 kinds of components were identified (Table 2). We found that amino acids, polysaccharides, aromatic acids, flavones, monoterpene glycosides, and others widely existed in MSJZD.

**TABLE 1:** Putative identification of MSJZD in positive ion mode.

NO.	Component name	Area	Retention time	Formula	Precursor Mass	Found at Mass	Mass error (ppm)	Library score	Isotope ratio difference
1	L (+)-Arginine	6674000	1.11	C6H14N4O2	175.119	175.1184	−3.3	89.1	0.8
2	Glutamic acid	299100	1.16	C5H9NO4	148.06	148.0602	−1.4	97.1	1.5
3	Betaine	296500	1.17	C5H11NO2	118.086	118.0861	−1.1	100	0.8
4	Trigonelline	1389000	1.21	C7H7NO2	138.055	138.0545	−3.5	96.9	1.8
5	Proline	5169000	1.23	C5H9NO2	116.071	116.0703	−2.4	98.8	1.3
6	Cytidine	61590	1.28	C9H13N3O5	244.093	244.0925	−1.3	100	3.7
7	Pipecolinic acid	792900	1.32	C6H11NO2	130.086	130.0862	−0.7	77.4	0.6
8	Adenine	800400	1.66	C5H5N5	136.062	136.0615	−2.1	97.6	2.4
9	Nicotinic acid	172900	1.74	C6H5NO2	124.039	124.0391	−1.5	99.8	1.2
10	Nicotinamide	231600	1.86	C6H6N2O	123.055	123.0551	−1.6	98.6	1
11	6-Hydroxypurine	79120	1.93	C5H4N4O	137.046	137.0457	−0.7	79.4	1.6
12	Adenosine	1537000	3.31	C10H13N5O4	268.104	268.1036	−1.8	100	4.1
13	10-Deacetylasperulosidic acid	58800	3.35	C16H22O11	408.15	408.1503	0.6	95.8	2.6
14	Cordycepin	86000	3.39	C10H13N5O3	252.109	252.1089	−0.7	100	4.6
15	Guanosine	433700	3.45	C10H13N5O5	284.099	284.0988	−0.6	99.1	4.9
16	Phenylalanine	659900	4.16	C9H11NO2	166.086	166.0859	−2.1	99.8	1.7
17	Deacetyl asperulosidic acid methyl ester	217100	4.96	C17H24O11	422.166	422.1656	−0.3	99.4	8.4
18	Complanatoside	7232	5.52	C28H32O16	625.176	625.1759	−0.6	100	12.1
19	Asperuloside	30430	5.71	C18H22O11	415.123	415.1232	−0.7	87.4	1.9
20	Syringin	5659	6	C17H24O9	390.176	390.1753	−1.5	93.9	11.2
21	Chlorogenic acid	127500	6	C16H18O9	355.102	355.1021	−0.7	99.6	6.2
22	Vitamin B2	56030	6.82	C17H20N4O6	377.146	377.1454	−0.3	93.9	5.8
23	Isoquercitrin	84680	7.23	C21H20O12	465.103	465.1028	0.1	98.7	3.5
24	Quercetin	331500	7.23	C15H10O7	303.05	303.0499	−0.2	97.2	5
25	Hyperin	84680	7.23	C21H20O12	465.103	465.1028	0.1	99.1	3.5
26	Schaftoside	319500	7.29	C26H28O14	565.155	565.1555	0.6	87.3	9.6
27	Daidzin	29060	7.66	C21H20O9	417.118	417.1182	0.4	100	6.2
28	Rutin	358200	7.98	C27H30O16	611.161	611.1604	−0.4	97.2	10.9
29	Calycosin-7-O-glucoside	2881000	8.1	C22H22O10	447.129	447.1283	−0.6	100	9.4
30	Liquiritigenin	7249000	8.28	C15H12O4	257.081	257.0806	−1	93.8	4.8
31	Scopoletin	166200	8.29	C10H8O4	193.05	193.0494	−0.7	94.2	0.5
32	Peimisine	798300	8.83	C27H41NO3	428.316	428.3155	−1.1	89.1	9.8
33	Peimine	5136000	9.17	C27H45NO3	432.347	432.3468	−0.9	100	11.3
34	Pratensein-7-O-glucoside	56730	9.3	C22H22O11	463.123	463.1237	0.4	95.2	6.5
35	Naringenin	174900	9.42	C15H12O5	273.076	273.0756	−0.7	99.4	5.8
36	Peiminine	3702000	9.54	C27H43NO3	430.332	430.3312	−1	100	8.5
37	Ononin	2887000	10.38	C22H22O9	431.134	431.1333	−0.7	98.6	8.4
38	Berberine	118700	10.66	C20H17NO4	336.123	336.1231	0.2	97	8.8
39	Farrerol	747800	10.96	C17H16O5	301.107	301.1069	−0.6	81.1	6.5
40	Calycosin	1548000	11.17	C16H12O5	285.076	285.0756	−0.5	95.4	6.2
41	Isomucronulatol	89900	11.24	C17H18O5	303.123	303.1226	−0.2	91.7	4.5
42	Isomucronulatol-7-O-glucoside	25400	11.24	C23H28O10	465.176	465.1756	0.2	78.6	4.9
43	Astragaloside Ⅳ	25030	13.85	C41H68O14	785.468	785.4668	−1.7	92.5	7.5
44	Glycyrrhizic acid	11430000	14.23	C42H62O16	823.411	823.4098	−1.5	98.6	15
45	Parthenolide	89170	16.6	C15H20O3	249.149	249.1483	−0.8	71.6	4.9
46	Glabridin	30850	18.1	C20H20O4	325.143	325.1431	−1.1	96.2	3.5
47	Alantolactone	324600	18.55	C15H20O2	233.154	233.1532	−1.8	94.4	5.2

“jianguoyun” https://www.jianguoyun.com/p/DUoWHt0Q8-mCChiI5poE

**TABLE 2:** Putative identification of MSJZD in negative ion mode.

NO.	Component name	Area	Retention time	Formula	Precursor Mass	Found at Mass	Mass error (ppm)	Library score	Isotope ratio difference
1	Histidine	46050	1.08	C6H9N3O2	154.062	154.0623	0.4	96.3	1.6
2	L (+)-Arginine	217900	1.1	C6H14N4O2	173.104	173.1042	−0.9	90.5	2.9
3	Glutamic acid	46660	1.14	C5H9NO4	146.046	146.0459	0.1	93.7	1.1
4	Quinic acid	694400	1.23	C7H12O6	191.056	191.0559	−1.4	93.8	2.9
5	Maleic acid	469200	1.34	C4H4O4	115.004	115.0036	−0.4	99.6	0.5
6	Citric acid	1466000	1.81	C6H8O7	191.02	191.0194	−1.5	98.6	1.6
7	Amber Acid	125800	2.65	C4H6O4	117.019	117.0192	−0.8	93.5	1.1
8	Isoleucine	64480	3.04	C6H13NO2	130.087	130.0873	−0.5	100	0.3
9	Adenosine	21060	3.31	C10H13N5O4	266.089	266.0894	−0.2	96.5	4.2
10	Guanosine	636700	3.46	C10H13N5O5	282.084	282.0837	−2.3	98.7	6.3
11	10-Deacetylasperulosidic acid	3209000	4.02	C16H22O11	389.109	389.1084	−1.3	97.9	7.6
12	Phenprobamate	184000	4.18	C9H11NO2	164.072	164.0715	−1.3	95.6	3.5
13	L-Tryptophan	127100	5.39	C11H12N2O2	203.083	203.0825	−0.5	91.5	2.4
14	Chlorogenic acid	559600	6.01	C16H18O9	353.088	353.0874	−1.3	100	7.6
15	Asperuloside	25180	6.47	C18H22O11	413.109	413.1086	−0.8	94.7	2.9
16	Fraxin	4046	6.48	C16H18O10	369.083	369.0825	−0.7	83	17.9
17	Caffeic acid	223500	6.59	C9H8O4	179.035	179.0348	−1.1	82.1	4.1
18	Vitamin B2	14220	6.82	C17H20N4O6	375.131	375.1307	−0.8	99.2	2.6
19	Sibiricose A5	24920	6.88	C22H30O14	517.156	517.1558	−0.8	94.6	8.2
20	Pratensein-7-O-glucoside	14210	7.21	C22H22O11	461.109	461.1086	−0.6	98.1	3.7
21	Schaftoside	278000	7.29	C26H28O14	563.141	563.1402	−0.7	97.9	11
22	Daidzin	27360	7.67	C21H20O9	415.103	415.1029	−1.3	92.3	5.3
23	p-Coumaric acid	1381000	7.81	C9H8O3	163.04	163.0398	−1.6	99.2	4.3
24	Rutin	813500	7.98	C27H30O16	609.146	609.1454	−1.2	95.5	11.6
25	Calycosin-7-o-glucoside	1831000	8.1	C22H22O10	491.119	491.1184	−2.2	99.2	7.1
26	Isoquercitrin	83380	8.28	C21H20O12	463.088	463.0878	−0.8	89.7	1.4
27	Liquiritin	14010000	8.31	C21H22O9	417.119	417.1183	−2	97.5	9.1
28	Ferulic Acid	76560	8.38	C10H10O4	193.051	193.0505	−0.7	78.8	1.4
29	Isochlorogenic acid A	47060	9.33	C25H24O12	515.119	515.1187	−1.6	94.3	7.5
30	4-Hydroxybenzoic acid	230900	10.08	C7H6O3	137.024	137.0242	−1.6	94.3	1.3
31	Rhein	2069	10.18	C15H8O6	283.025	283.0257	−3.1	70	6.7
32	Ononin	2710000	10.39	C22H22O9	475.125	475.1235	−2.4	99.3	10.9
33	Liquiritigenin	1471000	10.83	C15H12O4	255.066	255.0655	−2.9	91.2	6.8
34	Quercetin	420300	11.1	C15H10O7	301.035	301.0345	−2.9	96.2	6.9
35	Calycosin	1436000	11.17	C16H12O5	283.061	283.0604	−2.8	96.1	7.9
36	Isomucronulatol-7-O-glucoside	504300	11.24	C23H28O10	463.161	463.1599	−2.3	95.7	9.4
37	3-Hydroxy-9,10-Dimethoxypterocarpan	120600	11.74	C17H16O5	299.092	299.0919	−1.9	83.1	5.6
38	Naringenin	67970	12.29	C15H12O5	271.061	271.0608	−1.5	97.2	5.4
39	Dihydroartemisinin	3205	12.8	C15H24O5	283.155	283.1549	−0.7	86.3	5.2
40	Astragaloside Ⅳ	152700	13.86	C41H68O14	829.459	829.4573	−2.1	98.8	17
41	Glycyrrhizic acid	22010000	14.25	C42H62O16	821.397	821.3945	−2.4	95.5	14.7
42	Astragaloside Ⅱ	852900	14.85	C43H70O15	871.47	871.4677	−2.3	96.9	17
43	Asiatic acid	29610	16.57	C30H48O5	487.343	487.3423	−1.2	100	3.2
44	Astragaloside I	966200	16.68	C45H72O16	913.48	913.4781	−2.4	99.7	17.3
45	Glabridin	97900	18.11	C20H20O4	323.129	323.1285	−1.1	87	9.4

### TGF-β1 Induced Cell Morphological Changes and EMT

**FIGURE 2:**
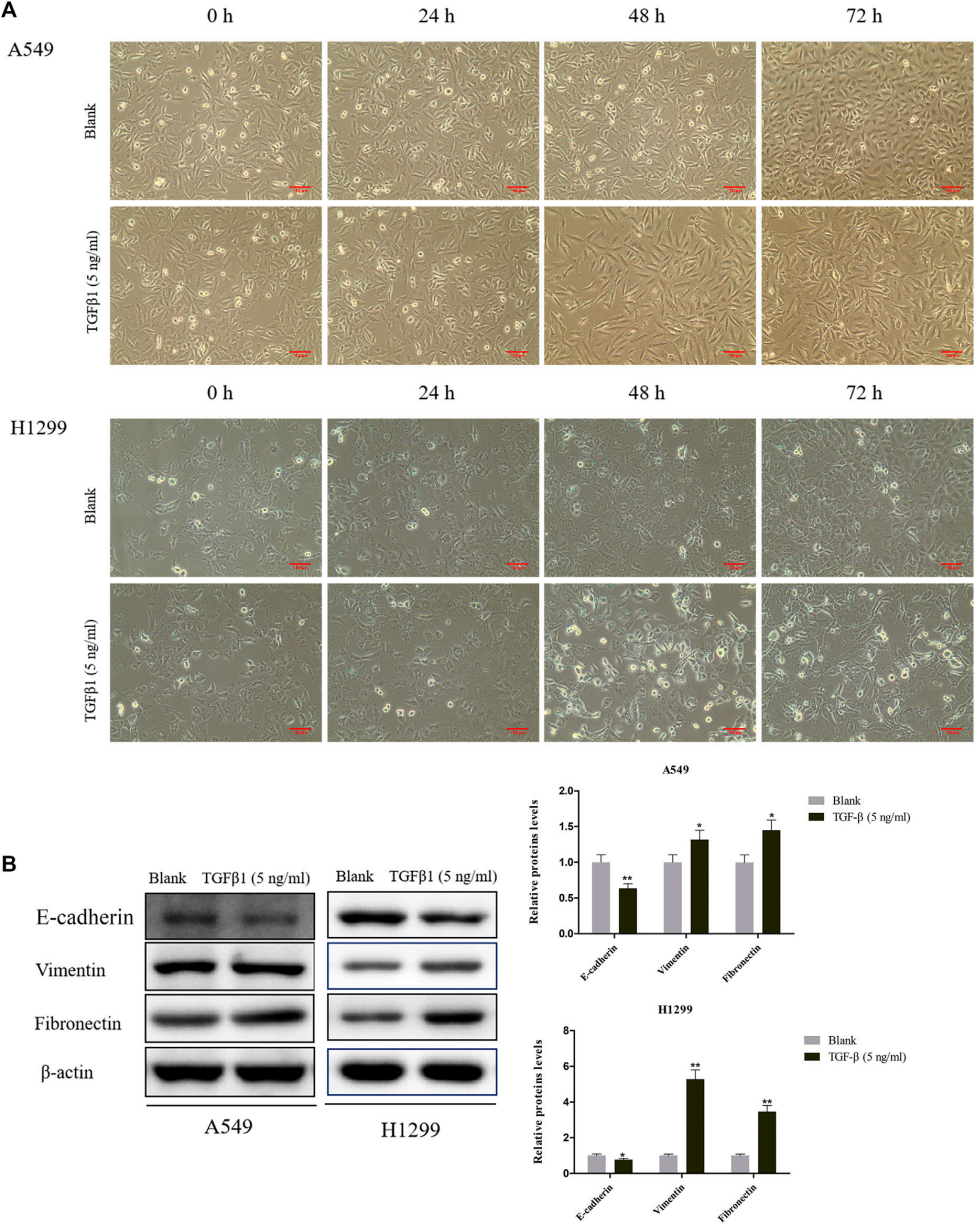
Effects of TGF-β1 on cell morphological changes and epithelial-mesenchymal transition of A549 and H1299 cells. **(A)** Microscopic photo of the lung cancer cells A549 and H1299 that treated with transforming growth factor-β1 (TGF-β1, 5 ng/ ml) for 0 h, 24, 48, and 72 h. **(B)** The levels of E-cadherin, Vimentin, and Fibronectin in A549 and H1299 cells treated with TGF-β1 (5 ng/ ml) for 72 h, as determined by western blot. *p < 0.05, **p < 0.01. versus the Blank group.

Next, we utilized TGF-β1 (5 ng/ ml) to treat A549 and H1299 cells for 24, 48, and 72 h. We discovered that the cell morphology changed to different degrees at different times after induction by TGF-β1, especially the morphology of A549 and H1299 cell changed most obviously after 72 h, which showed that the epithelial cells dominated by cubes changed to the spindle and fusiform mesenchymal morphology (Figure 2A). To a certain extent, TGF-β1 led to the boost of Vimentin and Fibronectin levels, as well as the attenuation of E-cadherin in A549 and H1299 cells that were treated for 72 h (Figure 2B).

### Serum Containing MSJZD Weakened the Cell Viability and Augmented Apoptosis in NSCLC Cells Induced by TGF-β1

**FIGURE 3:**
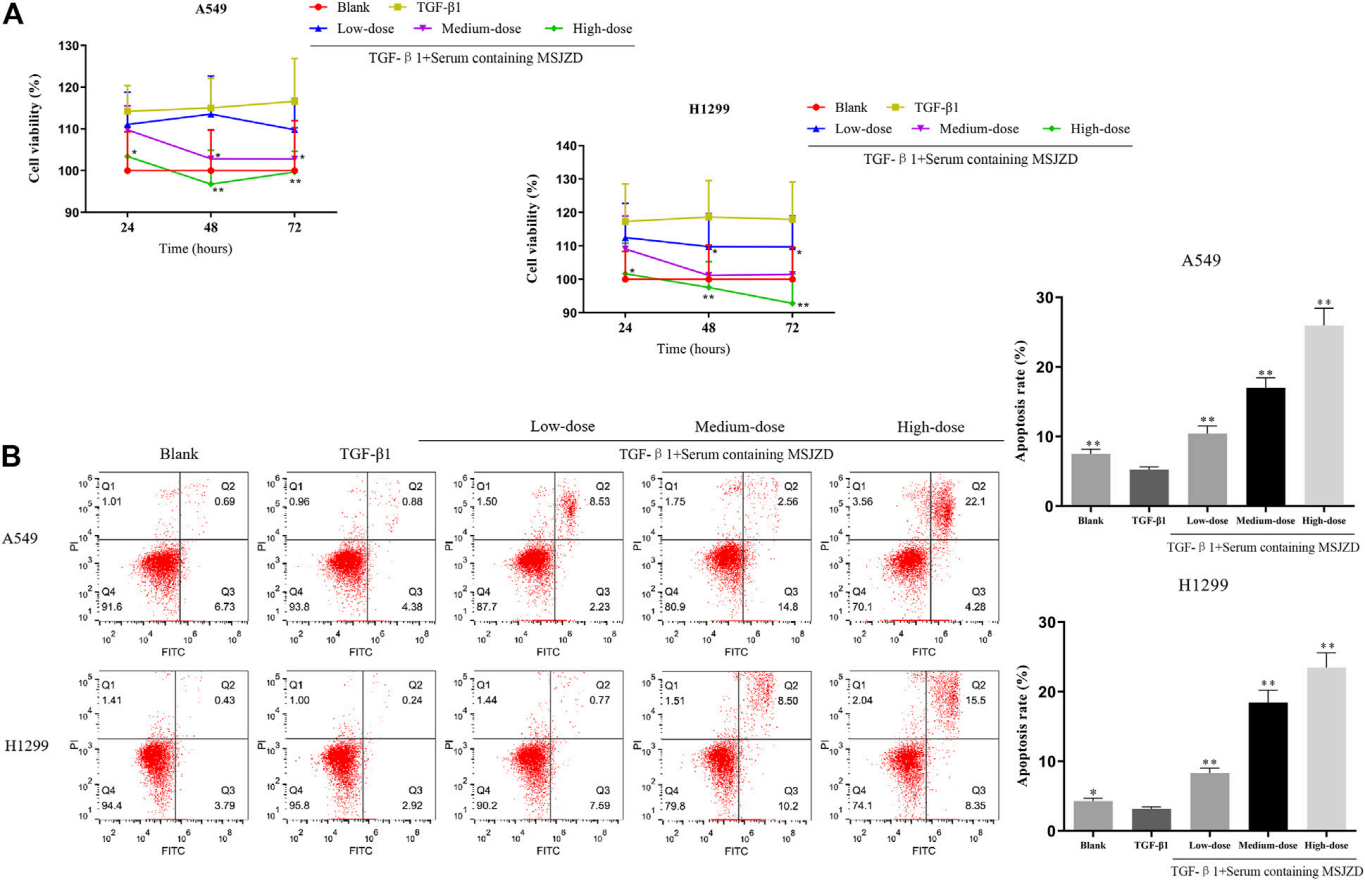
Serum-containing MSJZD weakened the cell viability and augmented apoptosis in NSCLC cells induced by TGF-β1. **(A)** Cell counting kit-8 (CCK-8) assay was performed in TGF-β1-induced A549 and H1299 cells treated with serum-containing MSJZD. **(B)** The A549 and H1299 cells were treated with blank control or serum-containing MSJZD and analyzed by flow cytometer to indicate cell apoptosis. *p < 0.05, **p < 0.01 vs. the TGF-β1.

In this work, to probe the role of serum-containing MSJZD in NSCLC cells triggered by TGF-β1, we conducted CCK-8 and flow cytometer assays. Cells were subjected to 5 ng/ ml TGF-β1 and 10% serum-containing MSJZD from different groups. Functionally, the results unveiled that TGF-β1 led to the boost of cell viability and the inhibition of apoptosis. Interestingly, serum-containing MSJZD evidently attenuated cell viability and induced apoptosis in TGF-β1-mediated NSCLC cells and the effect of high-dose was higher than medium-dose and low-dose (Figures 3A,B). These findings revealed that serum-containing MSJZD has an inhibitory effect in TGF-β1-mediated NSCLC cells.

### Serum Containing MSJZD Mitigated the Migration and Invasion in NSCLC Cells Triggered by TGF-β1

**FIGURE 4:**
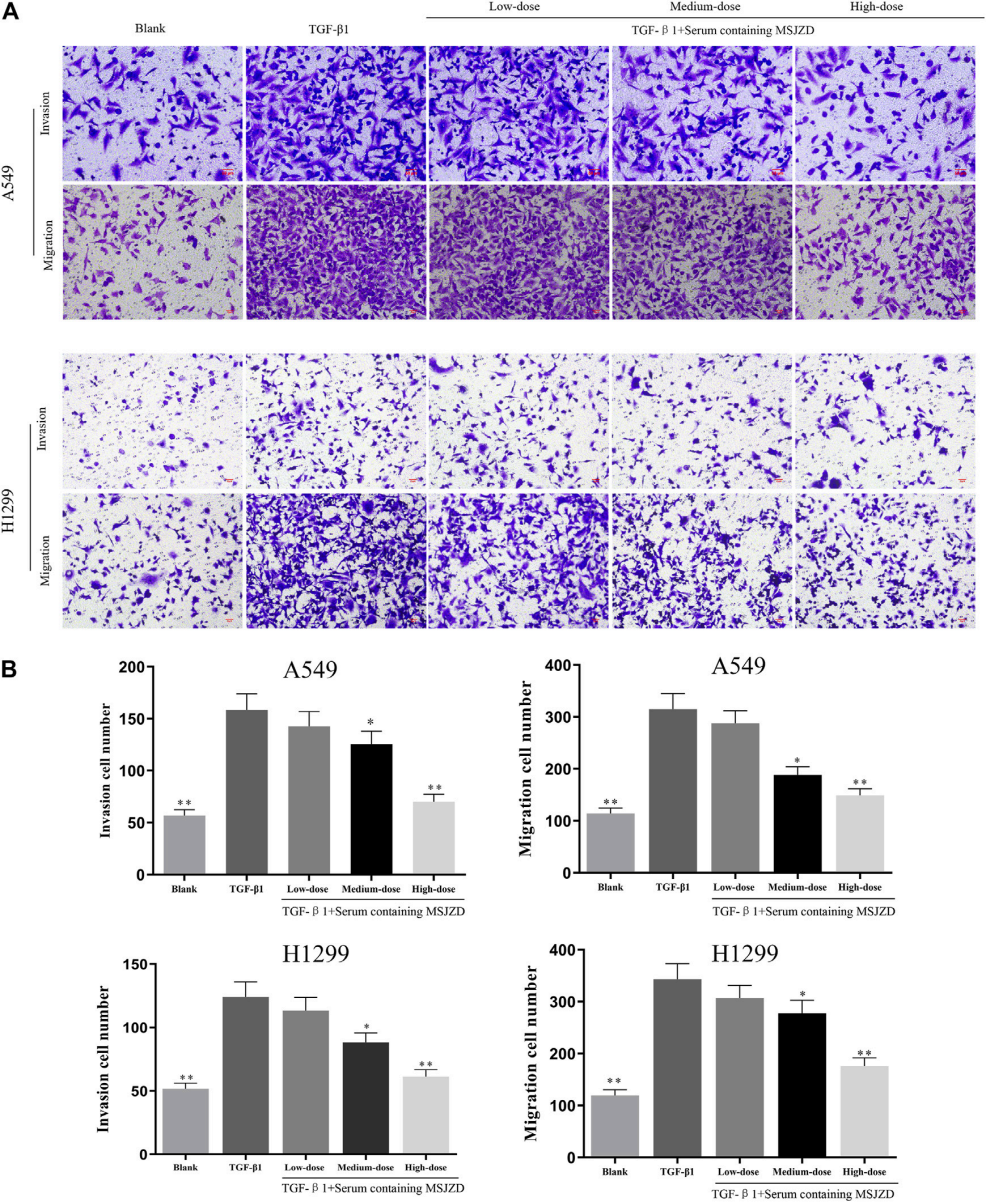
Serum-containing MSJZD mitigated the migration and invasion in NSCLC cells triggered by TGF-β1. **(A)** Transwell assays were conducted to examine the effects of serum-containing MSJZD on A549 and H1299 cell migration and invasion. **(B)** Statistical analysis of invasion and migration cell number in A549 and H1299 cells. *p < 0.05, **p < 0.01. versus the TGF-β1 group.

**FIGURE 5:**
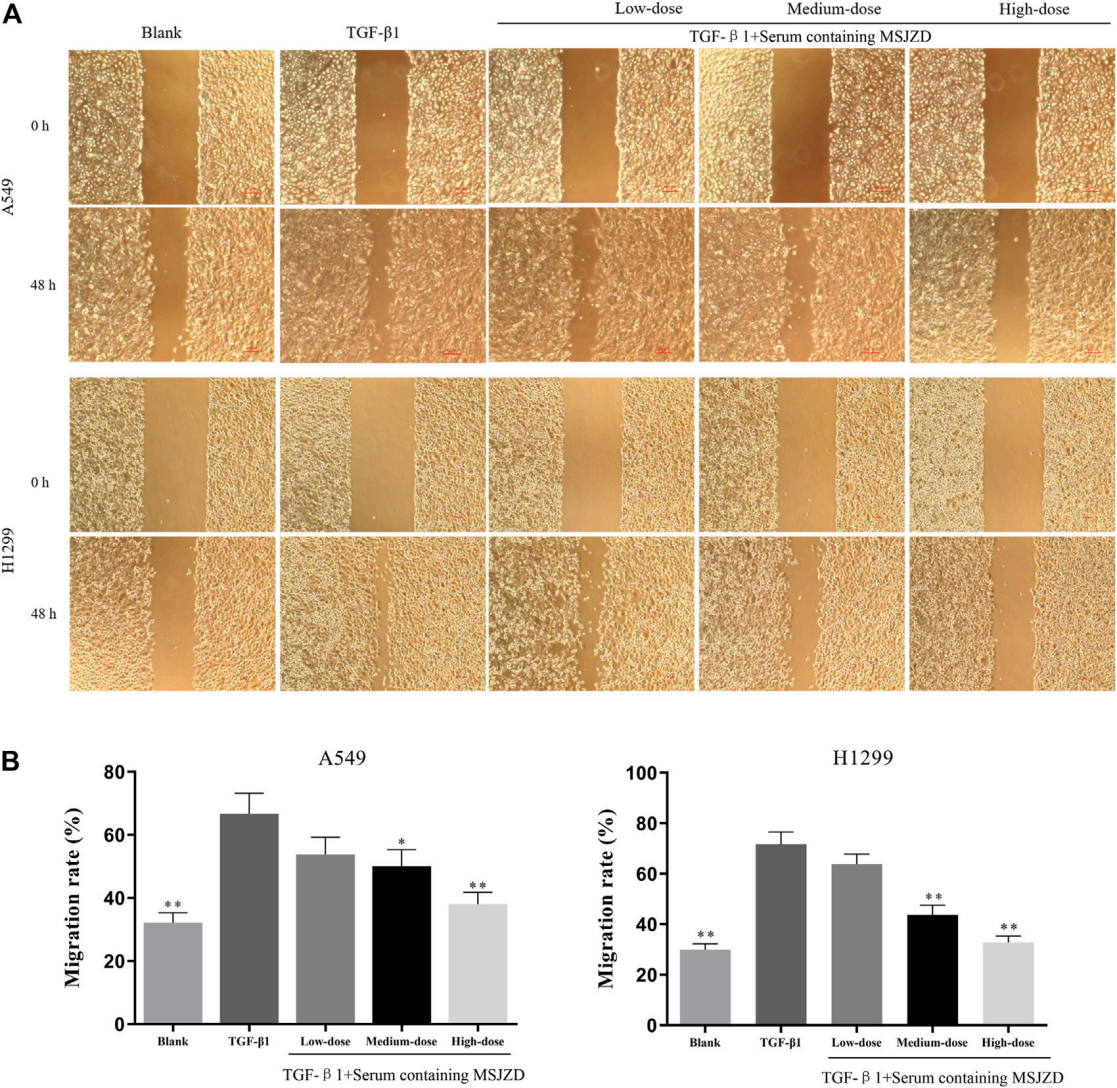
Effects of serum-containing MSJZD on the migration of NSCLC cells *in vitro*. Wound healing **(A)** and quantitative assay **(B)** were performed to evaluate the migration ability of NSCLC cells. *p < 0.05, **p < 0.01. versus the TGF-β1 group.

To check the function of serum-containing MSJZD on the migration and invasion in NSCLC cells triggered by TGF-β1, we utilized 5 ng/ ml TGF-β1 and different doses of serum-containing MSJZD to treat cells. As displayed in Figures 4, 5, we further proved that the migration and invasion of A549 and H1299 cells were extremely strengthened by TGF-β1 (*p* < 0.01). More importantly, we discovered that the enhanced effects were partially offset by serum-containing MSJZD in a dose-dependent way.

### Serum Containing MSJZD Weakened EMT, AKT/GSK3β Pathway and Induced Apoptosis-Related Markers in NSCLC Cells Triggered by TGF-β1

**FIGURE 6:**
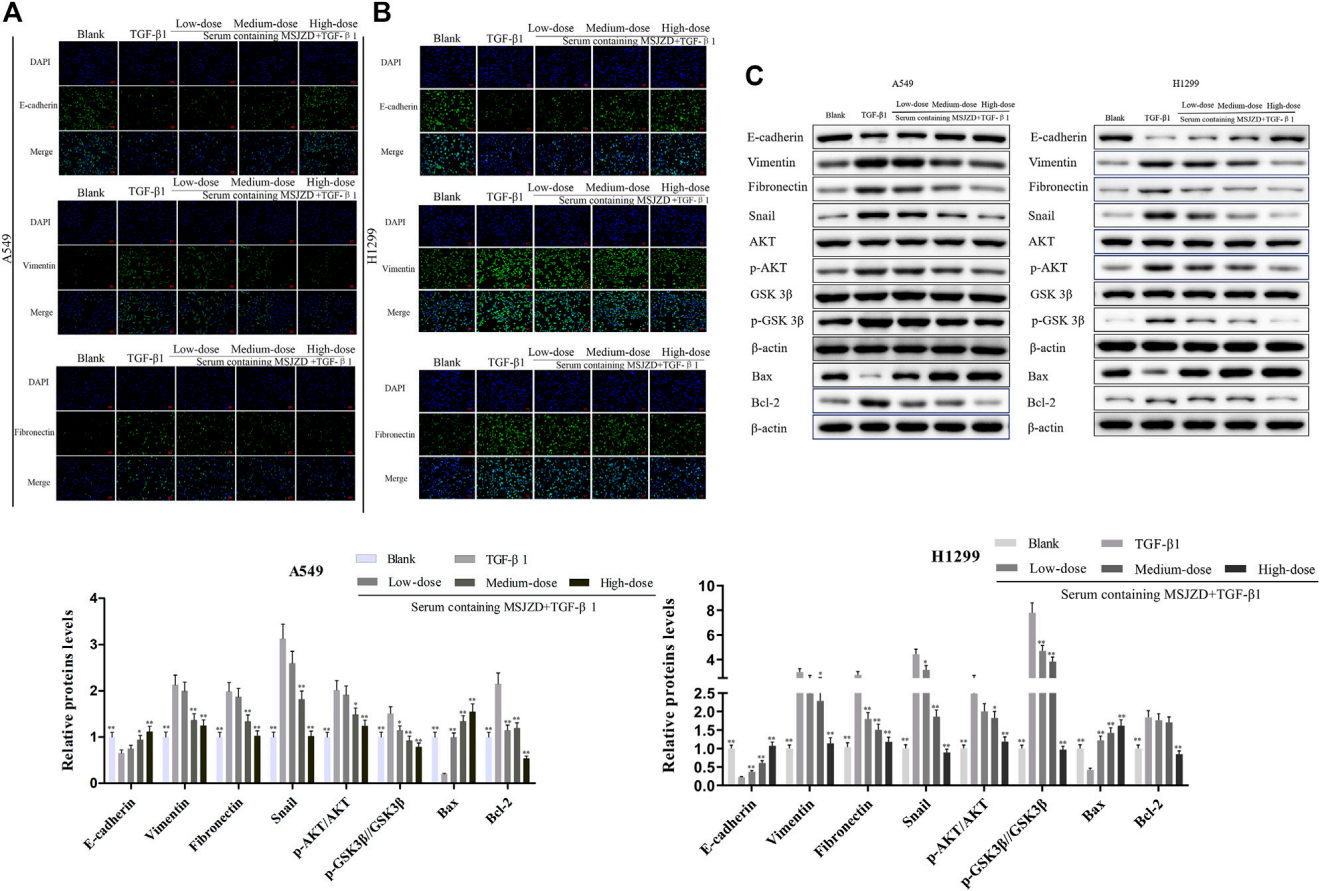
Serum-containing MSJZD weakened EMT, AKT/GSK3β pathway and induced apoptosis-related markers in NSCLC cells triggered by TGF-β1. The EMT protein levels of A549 **(A)** and H1299 **(B)** were detected by immunofluorescence. **(C)** The levels of E-cadherin, Vimentin, Fibronectin, Snail, p-AKT, AKT, p-GSK3β, GSK3β, and apoptotic proteins were detected by western blot after treatment with serum-containing MSJZD in A549 and H1299 cells. *p < 0.05, **p < 0.01. versus the TGF-β1 group.

Then, to identify the mechanism of MSJZD on NSCLC cells, we measured EMT-related factors by immunofluorescence and western blot assays. The positive expressions of Vimentin and Fibronectin were enhanced and the positive expression of E-cadherin was blunted by TGF-β1, whereas serum-containing MSJZD restrained the positive expressions of Vimentin and Fibronectin and augmented the positive expression of E-cadherin in TGF-β1-mediated NSCLC cells, and the influence of high-dose was higher than medium-dose and low-dose (Figures 6A,B). The results of western blot clarified that TGF-β1 caused the inhibition of E-cadherin and Bax and the boost of Vimentin, Fibronectin, Snail, p-AKT, p-GSK3β, and Bcl-2, while the above effects were overturned by serum-containing MSJZD (Figure 6C).

### MSJZD Attenuated the Tumor Growth, Promoted Histopathological Damage, and Induced Apoptosis in A549 Tumor-Bearing Nude Mice

**FIGURE 7:**
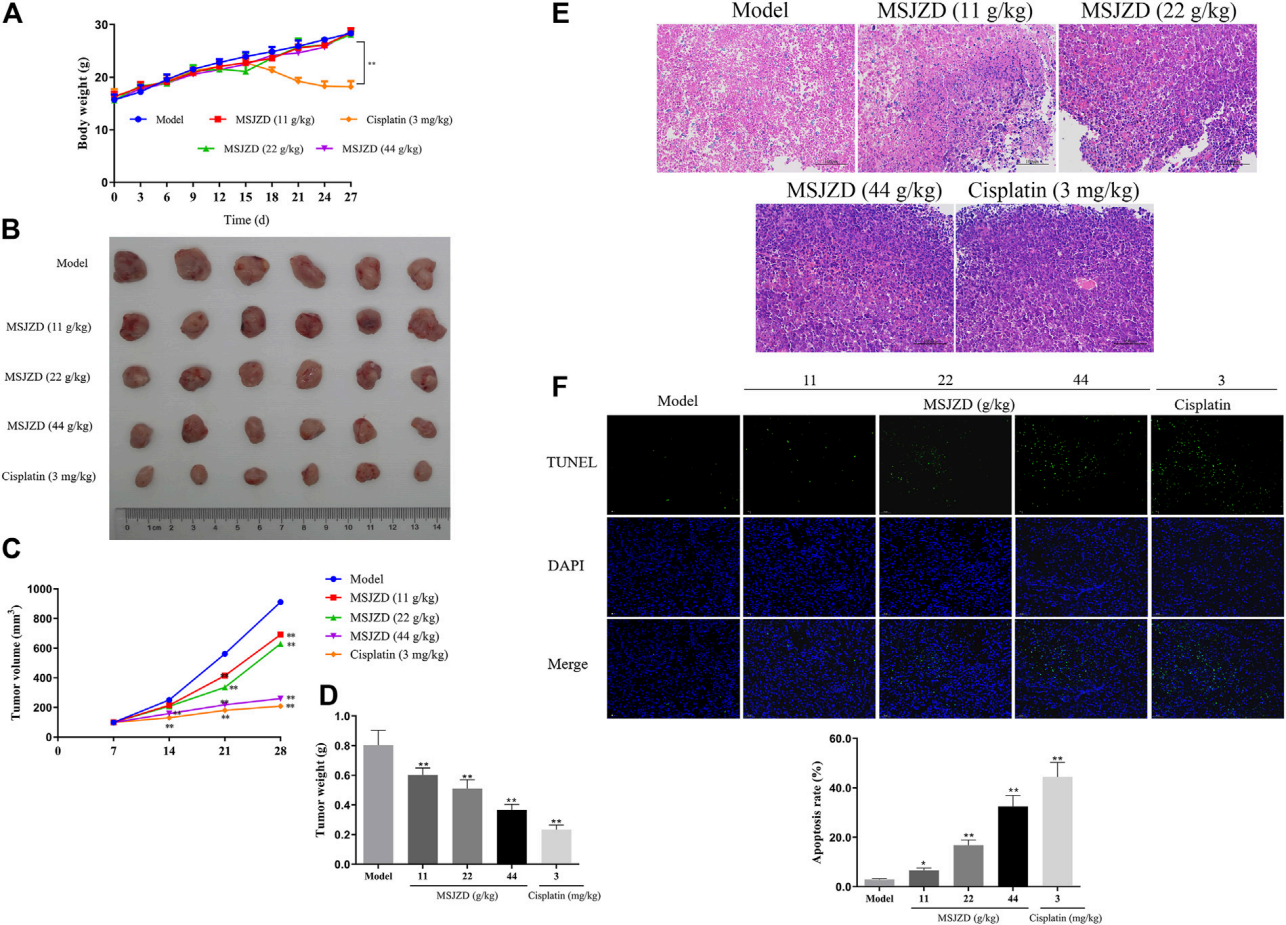
MSJZD attenuated the tumor growth, promoted histopathological damage, and induced apoptosis in A549 tumor-bearing nude mice. **(A)** Body weight of mice after MSJZD treatment was determined every 3 days. **(B)** Photograph of subcutaneous xenografts in A549 tumor-bearing nude mice after 28 days of treatment of MSJZD. The volume **(C)** and weight **(D)** of xenografts in MSJZD and Cisplatin groups. **(E)** Microscopic images of xenografts were observed by H&E staining. **(F)** TUNEL assay was performed to assess the apoptosis rate of xenografts in MSJZD and Cisplatin groups. *p < 0.05, **p < 0.01. versus the model group.

To further confirm the accuracy of *in vitro* experiments, we established an A549 cell tumor-bearing model in mice. MSJZD failed to affect mice’s body weight, while cisplatin treatment significantly reduced the mice’s body weight (Figure 7A). MSJZD restrained the tumor volume and weight in a dose-dependent way and the inhibitory role of cisplatin was the most obvious (Figures 7B–D). The H&E staining results illustrated that in the model group, the tumor tissue structure and cell morphology were complete (Figure 7E). In the high-dose MSJZD group and cisplatin group, the cell structure of tumor tissue was destroyed and the nucleus was seriously condensed. Moreover, TUNEL staining exhibited that MSJZD and cisplatin-induced apoptosis of A549 tumor-bearing nude mice and the promotion of 44 g/kg MSJZD on apoptosis was higher relative to the 11 and 22 g/kg MSJZD (Figure 7F).

### MSJZD Restrained EMT, AKT/GSK3β Pathway, and TGF-β1 Expression in A549 Tumor-Bearing Nude Mice

**FIGURE 8:**
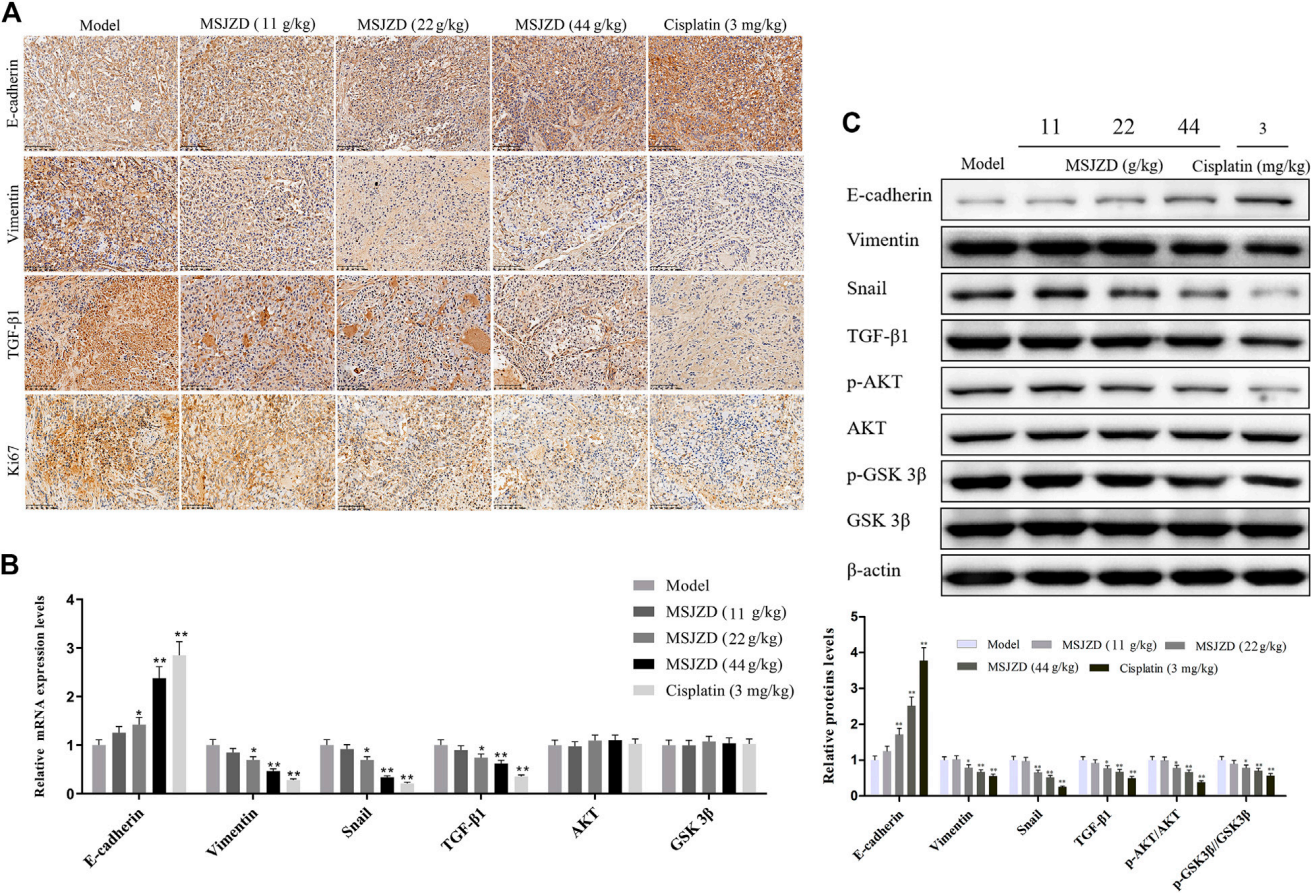
MSJZD restrained EMT, AKT/GSK3β pathway, and TGF-β1 expression in A549 tumor-bearing nude mice. **(A)** Immunohistochemical (IHC) staining of xenograft tumors. **(B)** The mRNA expression levels of E-cadherin, Vimentin, Snail, TGF-β1, AKT, and GSK3β in the experimental groups were determined by qRT-PCR. **(C)** The expression levels of E-cadherin, Vimentin, Snail, TGF-β1, p-AKT, AKT, p-GSK 3β and GSK 3β in the experimental groups were determined by western blot. *p < 0.05, **p < 0.01. versus the model group.

To verify the EMT-related protein (E-cadherin, Vimentin, Snail), TGF-β1, Ki67, and AKT/GSK-3β pathway associated protein expressions *in vivo*, IHC assay, qRT-PCR and western blot assays were performed in tumor tissues of mice. IHC assay proved that the positive expressions of Vimentin, TGF-β1, and Ki67 were inhibited by MSJZD in the dose-dependent way and cisplatin, while the positive expression of E-cadherin was enhanced by MSJZD and cisplatin (Figure 8A). Next, the qRT-PCR results illuminated that MSJZD and cisplatin augmented the E-cadherin level and weakened the Vimentin, Snail, TGF-β1 levels in A549 tumor-bearing nude mice (Figure 8B). Similarly, western blot revealed that E-cadherin expression in the MSJZD and cisplatin groups was significantly increased compared with the models. Additionally, western blot showed that the MSJZD and cisplatin markedly abolished the expression of Vimentin, Snail, TGF-β1, p-AKT, p-GSK 3β in A549 tumor-bearing nude mice, but MSJZD had no effect on the total protein expression levels of AKT and GSK 3β (Figure 8C). The regulation of 44 g/kg MSJZD on these genes was the most evident than 11 and 22 g/kg MSJZD.

## Discussion

Tumor metastasis is caused by the decrease of intercellular adhesion and the enhancement of tumor cell motility and invasiveness; the invasive characteristics enable cancer cells to separate from primary tumors and invade surrounding tissues through collective or individual cell migration (Otsuki et al., 2018). Modern medical research illustrated that EMT could give tumor cells the phenotype and cellular plasticity required for metastasis to acquire mesenchymal characteristics, thereby possessing high migration and invasion characteristics, and ultimately promote tumor cells to spread and metastasize (Lamouille et al., 2014; Marcucci et al., 2016). Many factors can induce the EMT process, including TGF-β1 (Eger et al., 2004). TGF-β1 is transforming growth factor β, which has multiple functions of modulating cell growth, apoptosis, differentiation, and migration, involving multiple signal pathways (Moustakas and Heldin, 2012). A clinical study in NSCLC exhibited that the positive expression rate of TGF-β1 in adenocarcinoma *in situ* (AIS) was 27.3%, and in minimally invasive adenocarcinoma (MIA) was 65.2%, demonstrating that TGF-β1 overexpression makes the tumor more invasive (Imai et al., 2013). TGF-β1 induced EMT in lung cancer cells, resulting in loss of cell polarity, decreased expression of epithelial marker E-cadherin, and increased expression of mesenchymal marker Vimentin (Massagué, 2008). Additionally, TGF-β 1-induced tumor cell development EMT is a classic pathological model used in the experimental study of cancer cell metastasis. *In vitro* studies, TGF-β1 induced EMT in NSCLC A549 cells, resulting in morphological changes, phosphorylation of Smad2 and Smad3, down-regulation of E-cadherin and up-regulation of Vimentin, N-cadherin, Snail, Slug, and MMP2 (Zhao et al., 2015; Feng et al., 2017; Lim et al., 2017). In this study, A549 and H1299 cells of NSCLC were used and induced by TGF-β1 for 72 h. Significant morphological changes occurred in the cells, including long spindle shape, discrete, and disappearance of intercellular adhesion. At the same time, the expression of cell epithelial protein E-cadherin was down-regulated, and the expression of interstitial proteins Vimentin and Fibronectin increased notably, proving that the EMT process occurred in NSCLC cells.

TCM has a unique advantage in anti-tumor metastasis and has been valued and affirmed by the medical community. The results have clarified that TCM compounds, such as Jiedu Sangen Decoction, Jianpi Yangzheng Xiaozheng Decoction, and Bu-Fei decoction, could weaken EMT to play the pharmacodynamic role of anti-tumor metastasis (He et al., 2017; Wu et al., 2019; Shan et al., 2020). In the current study, the constituents of MSJZD were analyzed using the UPLC-Q-TOF-MS method. Amino acids, polysaccharides, aromatic acids, flavones, and monoterpene glycosides may aid in the anti-cancer effects of MSJZD. Among them, Engelen et al. found that an impaired endogenous arginine synthesis was related to the reduced systemic arginine availability and NO synthesis in advanced NSCLC, and a dietary amino acid mixture is able to restore systemic arginine availability in cancer (Engelen et al., 2016). Also, chlorogenic acid, an ester with various pharmacological effects, is important in cancer therapy, including NSCLC (Hongtao et al., 2018). Moreover, Liao et al. reported that the combination of cordycepin and apatinib has a synergistically anticancer effect on NSCLC cells by down-regulating VEGF/PI3K/Akt signaling pathway (Liao et al., 2020). This result indicated that the constituents of MSJZD could be a promising drug against NSCLC.

Meanwhile, we evaluated the effects of serum-containing MSJZD in NSCLC A549 and H1299 cells-induced EMT by TGF-β1 for the first time. The results of cell function experiments illustrated that serum-containing MSJZD weakened the cell viability, migration, invasion, and augmented apoptosis in NSCLC cells induced by TGF-β1, exhibiting that MSJZD has a certain anti-NSCLC effect.

Invasion and metastasis are not only the important causes of death in NSCLC patients but also are the most intractable problems in NSCLC treatment. EMT plays a pivotal role in the metastasis of NSCLC (Mittal, 2016). EMT mainly involves multiple biological processes, such as the loss of cell-cell adhesion, the destruction of tumor basement membrane and extracellular matrix, and the reconstruction of the cytoskeleton, which plays a critical role in the metastasis of lung cancer (Chen et al., 2017). Tumor cells that occur in EMT usually undergo morphological and genetic changes. Morphological changes mainly include the evolution of cytokeratin structure from cubic epithelial cells to spindle-fusiform fibrocytes (Odero-Marah et al., 2018); gene changes include down-regulation of E-cadherin expression and up-regulation of Vimentin and Fibronectin expression (Li B et al., 2019). The above changes lead to the loss of cell-cell interaction, the loss of cell polarity, and the acquisition of interstitial cell “characteristics”, finally resulting in the stronger invasion of tumor cells (Konrad et al., 2020).

Changes in the expression of EMT-related markers are regulated by transcription factors such as Twist, Snail, and Zeb, which are activated in the early stage of EMT to coordinate the suppression of epithelial genes and the activation of the mesenchymal gene (Bai et al., 2017; Przygodzka et al., 2019). Therefore, blocking the occurrence of EMT is an effective treatment to limit the spread of tumor cells. In addition, the AKT/GSK3β pathway plays a major role in the EMT process (Xu et al., 2015). GSK3β is a downstream gene of PI3K/AKT signaling, which can phosphorylate Snail transcription factor to regulate EMT (Qiu et al., 2019). Snail is a kind of DNA binding protein containing zinc finger structure, which can recognize the E-box region upstream of the E-cadherin promoter, weaken the gene expression of E-cadherin, and promote the occurrence of EMT (Liu et al., 2014). It was demonstrated that in lung cancer, OLA1 modulated EMT through GSK3β/Snail/E-cadherin, thereby modulating the invasion and metastasis of lung cancer (Bai et al., 2016). Similarly, PI3K/AKT/GSK3β signaling has also been found to regulate EMT in breast and gastric cancer Zhang et al., 2017, Dai et al., 2016). Moreover, AKT/GSK3β signaling generated a critical role in the modulation of apoptosis in lung cancer cells (Li, Y et al., 2019). The boost of pro-apoptotic protein Bax and the reduction of anti-apoptotic protein Bcl-2 are the key factors to induce apoptosis (Liao et al., 2014). *In vitro* research, we discovered that TGF-β1 caused the inhibition of E-cadherin and Bax and the boost of Vimentin, Fibronectin, Snail, p-AKT, p-GSK3β, and Bcl-2, while the above effects were overturned by serum-containing MSJZD, exhibiting that MSJZD restrained EMT and induced apoptosis through the AKT/GSK3β pathway, thereby repressing the migration, invasion, and promoting apoptosis of NSCLC cells.

To further verify the accuracy of *in vitro* results, we established an A549 cell tumor-bearing model in mice. Our analysis manifested that MSJZD attenuated the tumor growth, promoted histopathological damage, and induced apoptosis in A549 tumor-bearing nude mice. More importantly, MSJZD augmented the E-cadherin level and weakened the Vimentin, Snail, TGF-β1, p-AKT, and p-GSK3β levels in A549 tumor-bearing nude mice, which was consistent with *in vitro* experiments. Although this study unveiled the anti-tumor mechanism of MSJZD to a certain extent, TCM prescription exerted its curative effect through multi-targets and multi-pathways. It should be noted that whether MSJZD may regulate EMT through other pathways, which needs more experimental verification. Meanwhile, the composition of MSJZD is extremely complex, and the active substance of its curative effect needs further studied and confirmed. In addition, Further establish animal models of spontaneous metastasis of lung cancer are needed to more intuitively evaluate the pharmacodynamics of MSJZD in modulating the EMT process of lung cancer and weakening tumor metastasis *in vivo*.

## Conclusion

In short, our research is the first one to demonstrate that MSJZD restrains EMT through the AKT/GSK3β pathway, thereby repressing the migration and invasion of NSCLC cells, which is expected to become a new therapeutic target for NSCLC.

## Data Availability

The raw data supporting the conclusions of this article will be made available by the authors, without undue reservation.
